# Retro-Odontoid Pseudotumor with Cervical Medullary Compression: A Case Report

**DOI:** 10.51894/001c.6768

**Published:** 2018-04-27

**Authors:** Wissam Elfallal, Samer Elfallal

**Affiliations:** 1 Beaumont Hospital System, Neurological surgery, PGY4 resident, Trenton, MI; 2 Beaumont Hospital System, Neurological surgery, Attending physician and Chief of Neurosurgery, Trenton, MI

**Keywords:** pannus, retro odontoid pseudo tumor

## Abstract

A retro odontoid pseudo tumor is a mass lesion lying posterior to the odontoid process along the dura. It is a disease process seen in inflammatory and non-inflammatory conditions causing chronic atlanto-axial instability. This type of mass has the potential to enlarge causing cervicomedullary compression and symptoms of myelopathy. In the past, authors have relied on a more invasive, direct approach to decompress the mass including an anterior trans oral odontoidectomy and a posterior trans-dural resection. The objective of this case report is to describe the use of an indirect approach, cervical fusion with decompressive laminectomy, to successfully treat a retro odontoid pseudo tumor in a geriatric patient. A male patient in his late 90’s presented with inability to ambulate, global paresis, and long tract signs in the upper extremities. He was found to have a large odontoid mass with compression at the cranio-cervical junction. He underwent cervical fusion with instrumented fixation from C1-6 and decompressive laminectomy from C4-6. Over a follow-up period of two years, there was improvement in the patient’s motor weakness and ambulation. Radiographic evaluation at the two-year mark showed marked reduction in pannus size. Indirect approaches to decompression in patients with retro odontoid pseudo tumor using techniques such as cervical fusion may be a safe for effective treatment in patients of advanced age, with multiple co-morbidities, and inability to tolerate lengthy surgical procedures.

## INTRODUCTION

Retro odontoid pseudo tumor is a disease entity characterized by a mass lesion lying posterior to the odontoid process (the upward protuberance of the C2 vertebrae, or *dens*) and resting along the dural sheath outside of the spinal cord. This retro odontoid mass may also be referred to as an odontoid pannus or phantom tumor.^1^ Over time, it may enlarge, causing cervicomedullary compression due to proximity to the spinal cord at the level where the cervical spinal cord and brainstem meet. This may lead to myelopathy, a clinical syndrome defined by neurologic deficit arising from the spinal cord. In this case, the neurological symptoms were from extrinsic compression of the spinal cord at the cervicomedullary junction.^2^

This clinical syndrome can comprise a diverse set of symptoms including sensory, motor, and dexterity changes of the upper and lower limb, unstable gait, and urinary retention due to chronic spinal cord compression.^3^ In rare instances (as seen in this case), there may be development of pannus-related cysts leading to further compression of the upper spinal cord and lower medulla.

In the past, authors have suggested treatment of the odontoid pannus through a trans-oral approach with direct removal and decompression.^4^ More recently, surgeons have trended towards upper cervical spinal fusion with or without laminectomy for indirect decompression and treatment of the pannus.^5^ Patients with inflammatory and non-inflammatory conditions causing chronic atlanto-axial instability including rheumatoid arthritis, Down syndrome, Os-odontoideum, and Morquio syndrome are at high risk for development of these retro-odontoid masses.^6^

The early recognition of cervical myelopathy in the setting of odontoid pseudo tumor along with prompt surgical evaluation can reduce morbidity and improve spinal cord compression outcomes. In addition, early intervention is crucial in helping patients preserve their baseline functional status.^7^

## METHODS

### Case Report:

A man in his late 90’s presented to the authors’ neurosurgical service with an inability to ambulate. Detailed neurologic exam revealed global paresis, or weakness, of the upper and lower extremity, and atrophy of the intrinsic hand muscles with long tract signs noted in the upper extremity. In addition, this patient had global hyperreflexia and lower extremity spasticity. The authors noted no lower cranial nerve palsies on his examination and his mental status was normal. Bedsores were found on his heels along with a sacral ulcer. Overall, the patient had poor physical health largely due to his lack of physical activity and wheelchair-bound state.

After his work-up was completed, the patient was found to have large odontoid pannus causing compression at the cranial cervical junction. We also noted a cyst associated with this pannus causing significant mass effect on the medulla (Figure 1 E). On MRI, this produced a T2 hyperintense signal at the medulla consistent with encephalomalacia, or medullary atrophy (Figure 1 A). The patient also had diffuse spondylosis, or degeneration, of the cervical spine along with central stenosis at the C4-5 and C5-6 level (Figure 1 C). Both an MRI of brain and CT angiogram were obtained and failed to reveal any other pathology. The posterior odontoid mass was found to be due to his rheumatoid arthritis.

**Figure 1 A:**
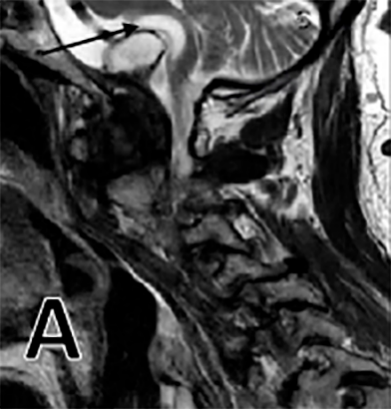
Pre-operative MRI sagittal T2 sequence, hyperintense signal (black arrow) within medulla representing encephalomalacia due to pannus compression.

**Figure 1 B:**
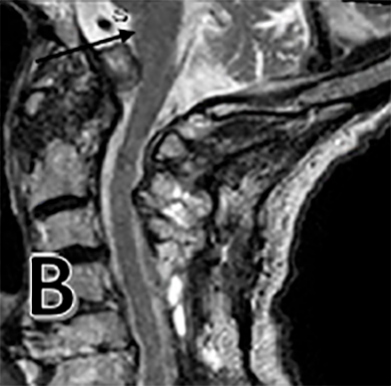
Post-operative T2 sagittal MRI showing reduction in cyst size and resolution of hyperintense signal within medulla (black arrow).

**Figure 1 C:**
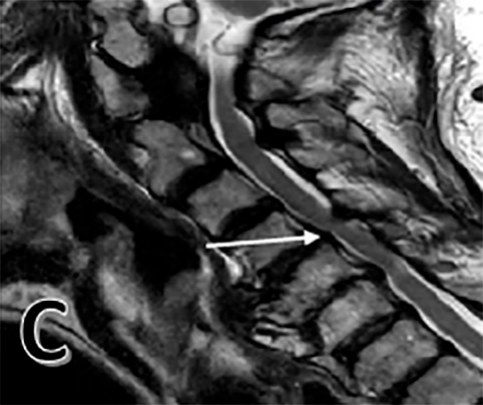
Pre-operative T2 sagittal MRI showing spondylosis and central stenosis from C4-6 (white arrow).

**Figure 1 D:**
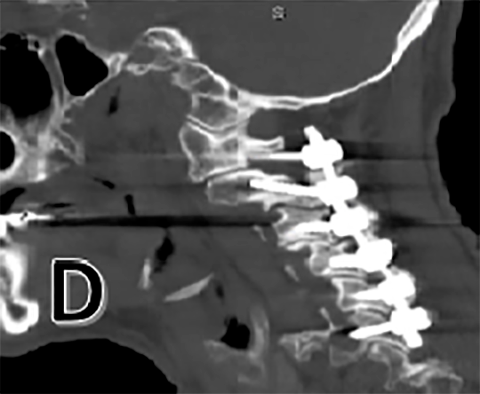
Post-operative sagittal CT scan Bone window showing C1-6 posterior cervical fusion.

**Figure 1 E:**
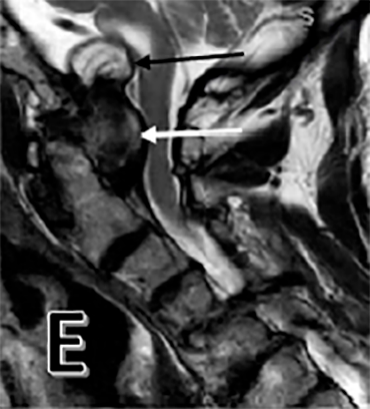
Pre-operative T2 sagittal with odontoid pannus (white arrow), and cystic component (black arrow).

**Figure 1 F:**
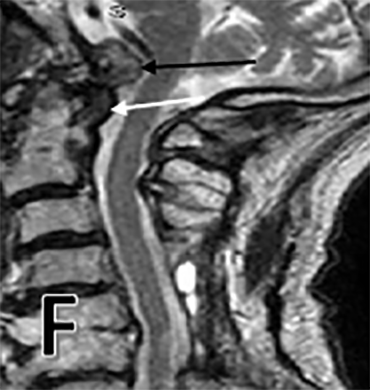
Post-operative T2 sagittal MRI showing odontoid process with reduction in pannus size (white arrow), and collapse of cystic component (black arrow).

### Interventions:

The authors had a long discussion with the patient and family in view of his advanced age and the risks of proceeding with surgery. Their primary surgical goal was to improve both his functional status and activities of daily living. Given his significant decline in functional status leaving him wheelchair-bound with high morbidity risks and poor quality of life, it was decided that the benefits of intervention outweighed the risks. The patient was medically optimized prior to surgery with full cardiac evaluation and clearance to decrease perioperative risk. His clinical presentation, physical exam findings, and pre-operative imaging were suggestive of compression of both the lower medulla and cervical spine and so we elected to complete a multi-level laminectomy. 

The patient would undergo posterior cervical arthrodesis, or fusion, with instrumented fixation from C1-6 with decompressive laminectomy (removal of the lamina to relieve pressure on the spinal cord and nerves) from C4-6 to treat associated stenosis, or narrowing of the spinal canal. Intraoperative neuromonitoring was utilized along with fluoroscopy for localization and lateral mass screw placement. The authors utilized an arterial line and a large bore 16-gauge intravenous catheter in the antecubital fossa to ensure adequate access for fluids and blood pressure monitoring. No signal changes were noted intraoperatively and patient was successfully extubated.

A post-operative CT scan is provided (Figure 1 D). The patient was placed in the ICU for post-operative monitoring. He tolerated the procedure well and his hospital course was uncomplicated. He was discharged in stable condition for a short course of inpatient rehabilitation.

## RESULTS

The patient was followed up for two years. There was improvement in his motor weakness symptoms and he was eventually able to ambulate without an assisted device. Radiographic evaluation at the two-year follow-up mark showed marked reduction in the pannus and associated cyst with resolution of T2 hyperintense signal within the medulla (Figures 1 B, F). While there are reports in the surgical literature of smaller-sized pannus causing cervical spinal cord compression, we have been unable to identify any reports of such a large odontoid pannus with associated cyst and medullary compression.^8^

## DISCUSSION

Retro odontoid pseudo tumor or odontoid pannus can be seen in inflammatory disease entities such as rheumatoid or psoriatic arthritis, and in the non-inflammatory settings (e.g., chronic dialysis, post-traumatic pseudo arthrosis, and degenerative disease).^5^ This mass sitting posterior to the odontoid process can be formed from different etiologies. The primary proposed mechanism is chronic atlanto-axial instability that leads to the development of this type of pannus. Authors have defined Atlanto-Axial instability using radiographic measurements of the atlas–dens interval (ADI). If the ADI is greater than 4 mm. with flexion on cervical spine x-rays, it is considered unstable.^9^ This instability leads to non-physiologic motion producing an inflammatory process with fibro cartilaginous mass formation in the posterior odontoid space which over time can lead to direct spinal cord compression.^10^ 

However, there are a few instances where no atlantoaxial instability is noted on imaging and yet there still may be development of this mass lesion.^9^ In our case, this patient had an underlying inflammatory spondyloarthropathy, but was under no active treatment. In general, the overall incidence of this condition is unknown, although a recent 11-year case series review found that incidence of retro odontoid pannus in patients with symptomatic atlanto-axial instability with associated risk factors including rheumatoid arthritis, os-odontoideum, dens fracture and Morquio syndrome was 23.2%.^6^

 In the past, authors have recommended a direct form of decompression with removal of the odontoid pannus. Surgical treatments included anterior trans oral odontoidectomy and posterior transdural resection. However, these surgical approaches have been associated with higher infection rates, spinal cord injury and in some instances reoccurrence of the mass.^11^ More recently, it has been proposed to surgically fuse the posterior spine, primarily at the C1 to C2 junction.

Authors have reported good radiographic and clinical outcomes one case report with complete resolution or reduction in mass size over a 45-month period, obviating the need for a transoral anterior approach.^5^ In this patient’s case, fusion not only included C1 and C2 vertebrae, but was extended to the lateral masses of C6. Decompressive laminectomy was completed at the C4 to C6 levels to treat the associated central canal stenosis. Our surgical intervention for this elderly man showed a good outcome with resolution of the pannus compression on the medulla and radiographic reduction of the mass.

We also elected this approach to reduce surgical risk and morbidity associated with surgery. The use of C1-2 fusion is a known safe treatment for atlanto-axial instability and has been extended as a treatment for painful arthritis.^12^ Some authors have even recommended occipital cervical fusion to treat associated occipital cervical instability and adjacent segment disease. This indirect treatment demonstrated radiographic improvement with good functional outcome.^10,13^ 

There is also literature supporting the use of C1 laminectomy for indirect decompression and reduction in pannus size. This strategy may be useful for patients unable to tolerate prolonged surgeries with significant blood loss and lengthy anesthesia exposure.^9^ There has been concern that C1 laminectomy without fusion may lead to further atlanto-axial instability. However, a reduction in neurologic symptoms can be feasibly obtained without progressive instability in most cases.^14^

## CONCLUSIONS

Retro odontoid pseudo tumors or odontoid pannus is a disease process characterized by formation of a posterior mass with possible cystic formation, which may lead to marked compression on vital neuronal structures at the cervico-cranial junction. Certain risk factors have been associated with an odontoid pannus including rheumatoid arthritis, Os odontoideum, or malunion of odontoid fracture. The basis of this disease process is believed to be due to atlanto-axial instability.

However, this may not be true for all cases, since it has been found that indirect techniques in the form of C1 laminectomy have helped reduce pannus size. In this patient’s case, we proposed cervical fusion and avoided a transoral approach due to his advanced age and inability to tolerate a more invasive procedure. After our two-year follow-up, the pannus and associated cyst had significant reduction with resolution of cord signal change within the medulla.

We therefore support the use of posterior fixation for treatment of this disease process, particularly in older patients. Further investigations of the pathophysiology of this disease process are warranted to help create a more unified treatment approach. Whichever surgical intervention is decided, early recognition and treatment of spinal cord compression can help improve neurologic outcomes.^7^

### Conflict of Interest

The authors declare no conflict of interest.
